# Optimization of Viability Treatment Essential for Accurate Droplet Digital PCR Enumeration of Probiotics

**DOI:** 10.3389/fmicb.2020.01811

**Published:** 2020-07-28

**Authors:** Anthony Kiefer, Peipei Tang, Samuel Arndt, Vincenzo Fallico, Connie Wong

**Affiliations:** DuPont Nutrition & Biosciences, Madison, WI, United States

**Keywords:** *Lactobacillus*, *Bifidobacterium*, enumeration, ddPCR, EMA, PMA, PE51, viability

## Abstract

Improvements offered by viability droplet digital PCR (v-ddPCR) include increased precision, specificity and decreased time to results making for an attractive alternative method to traditional plate count enumeration of probiotic products. A major hurdle faced in v-ddPCR, however, is distinguishing between live and dead cells. The objective of this study was to evaluate a combination of PMA and EMA (PE51) for viability treatment of freeze-dried probiotic powders. *Lactobacillus acidophilus* La-14 and *Bifidobacterium animalis* subsp. *lactis* Bi-07 were analyzed over a 2-log PE51 concentration gradient to investigate the efficiency across genus and assay targets. Results suggest a need to optimize viability dye concentration based on the genera of the organism, but also the assay target, even when analyzing the same organism. When optimized for PE51 concentration, strain specific v-ddPCR assays for both La-14 and Bi-07 were demonstrated to agree with plate count enumeration results. In conclusion, while these v-ddPCR assays require highly specific optimization, they are better suited for the future of the probiotic industry and are suggested to be implemented in probiotic product testing.

## Introduction

Scientific literature and clinical studies are increasingly showing the ability of certain gut-derived probiotic strains to benefit the host by supporting a healthy digestive tract and immune system (Hill et al., 2014; O’Bryan et al., 2013). Based on this, functional foods and dietary supplements containing bioactive probiotics have become one of fastest growing wellness categories, with a global market forecasted to be worth USD 64 billion by 2023 (GMI, 2016). This challenges the industry to produce vast quantities of probiotic products to be delivered to diverse geographical markets worldwide. To achieve this, probiotics are dried into highly concentrated powders as a convenient and inexpensive format facilitating both formulation into functional products as well as storage, handling and distribution without the need for specialized refrigerated containers (Muller et al., 2009).

It is known that probiotics can confer a health benefit on the host “when administrated in adequate amounts,” and that clinically proven functional attributes can only be associated with the specific strains and doses assessed in those studies (FAO/WHO, 2002; Hill et al., 2014). Consequently, regulatory authorities require manufacturers to provide data showing the ability of their probiotic formulations to systematically deliver health-promoting amounts of viable microorganisms at the time of consumption (Jackson et al., 2019). A generally accepted minimum dose is 1 billion viable bacteria per g of product (Hill et al., 2014), but often commercial preparations of probiotic strains need to recapitulate the doses of their clinical trials to support label claims (Fenster et al., 2019). This presents a major analytical challenge for the probiotics industry as enumerating and confirming the presence of the correct strains in clinically relevant viable doses becomes paramount to assessing the quality of commercial probiotic products.

The current probiotic industry standard uses traditional microbiological plate count methods to measure probiotic viability by promoting growth and colony formation on nutrient agar and rely on combinations of various media, selection supplements and culture conditions to enumerate specific genera and species. Culture-based methods, however, require long time-to-result (1–3 days), generate counts with large (15–30%) coefficient of variation (CV), and cannot differentiate closely related strains that share similar growth requirements and metabolic profiles (Corry et al., 2007; Davis, 2014; Vinderola et al., 2019). These major limitations call for better performing methods that can rapidly and accurately enumerate viable probiotics to the strain level and provide more reliable quality metrics for the industry.

Molecular methods offer the best alternative since rapid advances in genome sequencing and bioinformatics now enable detection and resolution of phylogenetically similar strains based on unique insertions/deletions or single nucleotide polymorphisms in DNA sequences (Briczinski et al., 2009; Hansen et al., 2018; Morovic et al., 2018). By combining *in silico*-designed strain-specific assays with polymerase chain reaction (PCR) quantitative approaches, rapid and accurate methods can be devised that greatly facilitate the selective enumeration and routine qualification of multi-strain products. Digital PCR (dPCR) represents the latest development in DNA quantification technologies and carries significant improvements over quantitative PCR as it is less sensitive to inhibitions (Huggett et al., 2013), shows a lower limit of detection (Qian et al., 2016) and does not require a standard curve for absolute quantitation (Hindson et al., 2011). dPCR partitions the DNA sample into either chip wells (cdPCR) or oil droplets (ddPCR) and uses a Poisson distribution to extrapolate absolute counts from the number of partitions showing amplification of single-copy gene targets (Hindson et al., 2011).

Recently, dPCR assays were shown to address the shortcomings of culture methods and were proposed as next-generation approaches to enumerate commercial probiotics. Using cdPCR in combination with strain-specific primers targeting single-copy genetic deletions, *Bifidobacterium animalis* subsp. *lactis* Bl-04 and *Lactobacillus acidophilus* NCFM were successfully differentiated against other strains of the same species and selectively enumerated when multiplexed (Hansen et al., 2018). Additionally, ddPCR was shown to enumerate several commercial probiotic strains with a high correlation to the gold standard of plate counts, and with the additional advantage of generating faster (4–8 vs. 24–72 h) and more accurate (1–3% vs. 12–20% CV) results (Hansen et al., 2020).

A major limitation of any PCR technology is, however, the inability to differentiate between viable and non-viable cells. To overcome this, Nogva et al. (2003) introduced the concept of viability PCR (v-PCR) where, prior to DNA extraction and amplification, samples are treated with a molecule that selectively enters cells with damaged membranes and intercalates to DNA. Light exposure irreversibly crosslinks the dye to the DNA, resulting in a permanent DNA modification that strongly inhibits its downstream amplification. Live cells with intact membranes generally exclude the dye and, as a result, their unmodified DNA is selectively amplified by PCR (Fittipaldi et al., 2012).

Ethidium monoazide (EMA) and propidium monoazide (PMA) are the DNA-intercalating dyes developed for v-PCR and several articles describe their individual use to quantify viable strains of lactic acid bacteria and probiotics (Fujimoto and Watanabe, 2013; Gobert et al., 2018; Kramer et al., 2009; Meng et al., 2010). Despite this, there is evidence that both PMA and EMA present limitations in their application as viability dyes.

Propidium monoazide is a monoazide derivative of propidium iodide (PI), which is widely used in flow cytometry as marker of dead cells. PI was found to surprisingly stain up to 40% of cells of environmental bacteria during exponential growth (Shi et al., 2007), whereas PMA underestimated heat-killed cells of *Listeria innocua* in comparison with plate counts (Løvdal et al., 2011).

Similarly, EMA is known to diffuse through intact membranes in some bacterial species and its accumulation to depend on the interplay between permeability and active efflux systems (Cawthorn and Witthuhn, 2008; Fittipaldi et al., 2012; Nikaido, 2001; Nocker and Camper, 2006). These findings questioned the notion that membrane integrity alone is a universal indicator of cell viability and prompted Codony et al. (2015) to suggest the simultaneous presence of an intact membrane and an active metabolism for cells to be considered viable. Their approach to assess cell functionality relied on the assumption that low concentrations of EMA will only accumulate in cells lacking the metabolic activity to expel the toxic molecule using ATP-driven efflux systems. Based on this, a patented combination of 50 μM PMA and 10 μM EMA was proposed to improve the accuracy of v-PCR by enabling the simultaneous measurement of membrane integrity and maintenance of internal homeostasis, respectively, and formed the basis for the commercial development of the PEMAX viability dye (Codony, 2016).

Despite this innovation in v-PCR technology, several other factors are known to come into play and affect the ability of v-PCR to differentiate between viable and non-viable organisms. Differential uptake of the same amount of EMA/PMA dye was observed for several gram-positive and gram-negative bacteria, and this was speculated to be related to species-specific differences in cell membrane and cell wall compositions. Furthermore, the length and sequence of the gene target appear to influence v-PCR results, as the efficiency of dye binding is probably dependent on the GC content and secondary structures of the target DNA (Chang et al., 2010; Fittipaldi et al., 2012; Nocker et al., 2006; Soejima et al., 2011).

We have previously shown that dPCR is a viable alternative to plate counts for the rapid, accurate and strain-specific enumeration of commercial probiotics (Hansen et al., 2018, 2020). In this study, we evaluate and demonstrate the need for optimizing the concentration of the dual stain PMA-EMA, here after referred to as PE51, based on the bacterial genus to be enumerated and on the gene to be targeted by viability droplet digital PCR (v-ddPCR).

## Materials and Methods

### Assay Design

**FIGURE 1:**
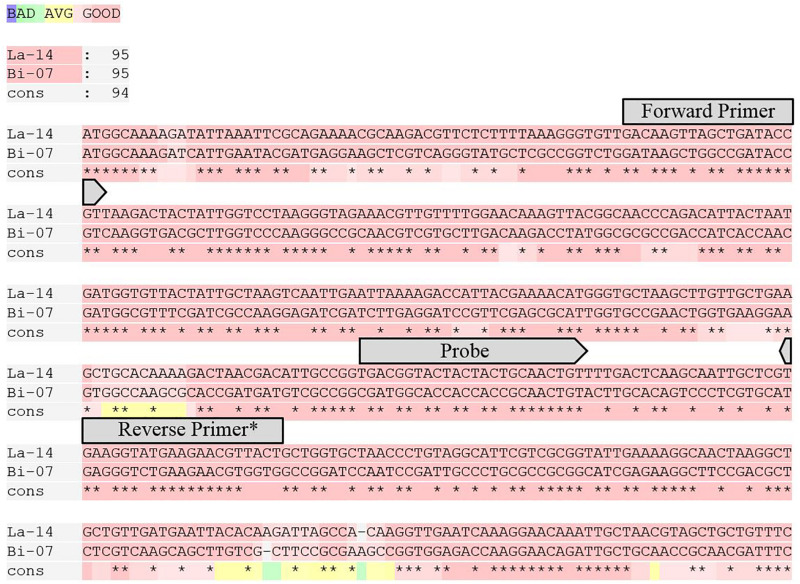
T-Coffee alignment of the first 450 bp of the GroEL gene for La-14 and Bi-07 (Notredame et al., 2000). Overlaid on the alignment are the locations of the primers and probes designed for this target. * For melting temperature purposes, the first two base pairs on the 5′ end of Reverse Primer were only utilized in La-14 GroEL assay.

Strain specific primer and probe assays were first reported by Hansen et al. (2020). GroEL assays designed for this study were in the same location on the GroEL gene for Bi-07 and La-14 and to have similar amplicon length, GC content, and Tm to strain specific counterparts (Figure 1 and Table 1). Genome analyses and assay designs were completed via Geneious 11.1.5 (Biomatters, Auckland, New Zealand), and assay analyses were performed with OligoAnalyzer Version 3.1 (IDT, Integrated DNA Technologies, Coralville, IA, United States).

**TABLE 1:** Strain specific and GroEL assays for La-14 and Bi-07.

**Assay name**	**Oligo Type**	**Sequence (5′-3′)**	**Tm (°C)**	**Amplicon size (bp)**	**GC (%)**
Bi-07 Strain Specific	Forward	TTC AAG CCG ACG TAC TTG CT	60	210	56.7
	Probe	/5HEX/CG AAG ATG A/ZEN/T GTC GGA ACA CAA ACA CCC GG/3IABkFQ/			
	Reverse	CGA GGC CAC GGT GCT CAT ATA GA			
Bi-07 GroEL	Forward	GAT AAG CTG GCC GAT ACC GT	60	262	55.0
	Probe	/5HEX/CG ATG GCA C/ZEN/C ACC ACC GCA ACT GT/3IABkFQ/			
	Reverse	CCA CGT TCT TCA GAC CCT CA			
La-14 Strain Specific	Forward	CCG GTT AAT AAA ATC TTT TCA CCT TG	56	202	35.6
	Probe	/56-FAM/AG TTG ATC A/ZEN/G TCA GCA AGT AGT GTT ATG G/3IABkFQ/			
	Reverse	GCA GTT ATT AAT CGT GAT TTG CAT ATA AAT T			
La-14 GroEL	Forward	GAC AAG TTA GCT GAT ACC GT	56	264	40.5
	Probe	/56-FAM/TG ACG GTA C/ZEN/T ACT ACT GCA ACT GT/3IABkFQ/			
	Reverse	AGT AAC GTT CTT CAT ACC TTC A			

### PE51 Solution

PE51 was created as a combination of 5-parts PMA to 1-part EMA based on the work performed by Codony et al. (2015). Molecular biology grade water (1.19 ml) was added to 5 mg of EMA (Biotium, Fremont, CA, United States) to create a 10 mM solution. PMA was ordered premixed as a 20 mM PMA in water solution (Biotium). PMA and EMA concentrations were verified on a Genesys 20 spectrophotometer (ThermoFisher, Waltham, MA, United States) at wavelengths of A470 and A464, respectively. Calculated concentrations were used to create a 500 μM PE51 solution composed of 5-parts PMA and 1-Part EMA with molecular biology grade water. A 50 μM concentration was also created by diluting 500 μM solution (1:10) with molecular biology grade water.

### Sample Preparation and Viability Treatment

Samples were prepared and treated based on previous literature (Hansen et al., 2018; Morovic et al., 2018) with some modifications outlined below.

Three random commercial lots of *Lactobacillus acidophilus* La-14 (L1, L2, and L3) and *Bifidobacterium animalis* subsp. *lactis* Bi-07 (B1, B2, and B3) were weighed (11 g) into Whirl Pak bags, then rehydrated in 99 ml of Remel Butterfield’s phosphate buffer (BPB) (Fisher, Hampton, NH, United States) creating a (1:10) dilution. Samples were serially diluted in BPB to a final concentration of 1:20,000 for viability treated samples (v-ddPCR) or 1:100,000 for untreated samples (t-ddPCR). Dilutions were calculated based upon known plate count enumeration results. Based upon these returned ddPCR copies/g results, samples designated for the linear analysis experiment were serially diluted to a theoretical count of 20–2000 copies/g or a 2-log concentration gradient,.

For v-ddPCR samples, 1.2 ml of diluted samples were transferred into 1.5 ml centrifuge vials, then treated with specified concentrations of PE51 spanning a 2-log concentration gradient. Additionally, concentrations of 170 and 1,400 nM, optimized concentrations for Bi-07 and La-14 strain specific assays, respectively, were included. These concentrations were chosen based on previous work comparing v-ddPCR and plate count enumeration (data not shown). Concentrations used were 42, 170, 210, 420, 1,400, 2,100, and 4,200 nM which equates to 1, 4, 5, 10, 32.5, 50 μl of 50 μM, and 10 μl of 500 μM PE51. Vials were gently vortexed, then placed into a 38°C incubator, protected from light and gently shaken at 200 RPM for 30 m to facilitate reaction. After incubation, samples were transferred to a PMA-lite LED Photolysis Device (Biotium) for 15 m. One ml of treated and untreated samples was then transferred into prefilled 2.0-ml tubes containing Triple-Pure high-impact 0.1-mm zirconium beads (D1032-01; Benchmark Scientific, Edison, NJ, United States) for cell lysis on Bead Ruptor Elite (Omni International, Kennesaw GA, United States) at 6.30 m/s for 1 m.

### ddPCR

**FIGURE 2:**
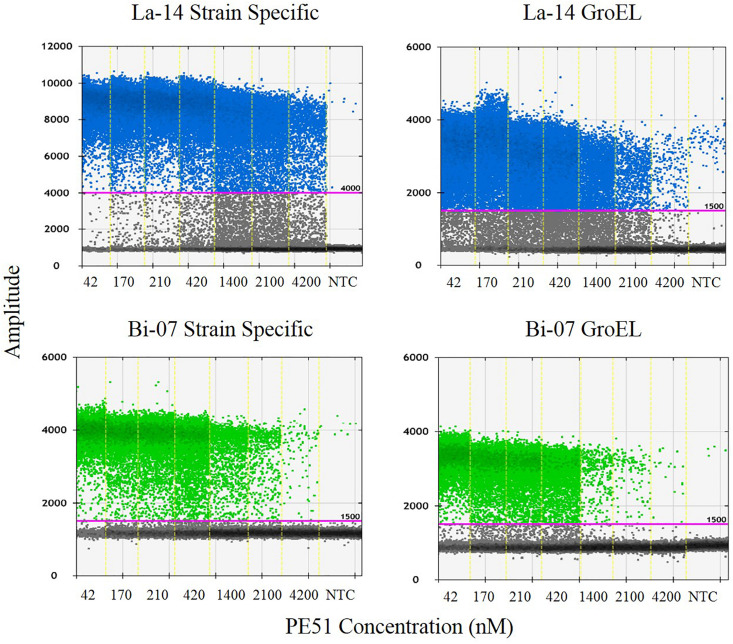
QuantaSoft output demonstrating specified thresholds and summarizing the results from each assay and concentration tested. Also included is a no template control (NTC) result for each assay.

Polymerase chain reaction reaction mixtures were created by combining the following reagents in the specified concentrations: 0.42 μL of molecular biology grade water, 4.5 μl of forward and 4.5 μl reverse primers (Integrated DNA Technologies (IDT), Coralville, IA, United States) (Table 1), 2.08 μl of probe (IDT) (Table 1), 12.5 μl of ddPCR Supermix for probes (no dUTP), and 1 μL of lysed sample. Twenty-five μl mixture was transferred to ddPCR 96-Well Plates in triplicate. Plates were sealed by PX1 PCR Plate Sealer with pierceable foil PCR plate heat seal and transferred to AutoDG where samples where partitioned into oil droplets with automated droplet generation oil for probes. After droplet generation, new plate was sealed and placed in C1000 thermal cycler under the following conditions: 95°C for 10 min, 95°C for 30 s, and 56/60°C (ramp rate 2.0°C/s) (La-14/Bi-07, respectively) for 1 m repeated for a total of 40 cycles, followed by 98°C for 10 min, then held at 10°C until transfer to a QX200 Droplet Reader. Droplets were analyzed utilizing QuantaSoft Software v. 1.7. An amplitude threshold for positive droplets was set at 4,000 fluorescence units for La-14 strain specific assay, and 1,500 fluorescence units for all other assays (Figure 2). Except for primers and probes, all other equipment, reagents and analytical software were from Bio-Rad (Pleasanton, CA, United States).

### Plate Counts

Plate count enumeration was performed in accordance with USP monographs for *Bifidobacterium animalis* subsp. *lactis* (USP-NF, 2017a) and *Lactobacillus acidophilus* La-14 (USP-NF, 2017b). For rehydration, samples of 11 *g* were added to 99 ml of MRS broth (BD Difco, Sparks, MD, United States) blended using Stomacher 400 Circulator at 230 RPM for 30 s or 2 min (Bi-07, La-14, respectively), held at room temperature for 30 min and then blended again as previously described. Samples were diluted 1:10,000,000,000 or 1E9 via serial dilution in 99 ml Flip-Top Dilution Bottles with Peptone Water (3M, Maplewood, MN, United States). One ml of diluted samples was transferred onto empty petri dishes in triplicate. Approximately 15 ml of 45°C MRS agar (BD) was poured into petri plates, plates were gently swirled to mix, then allowed to solidify at room temperature. Once solidified, plates were inverted, placed into anaerobic chambers with BD GasPak EZ sachets (BD), then incubated at 37°C for 48–72 h. Results were recorded as colonies visualized on plates after incubation and back calculated as colony forming units per gram of product (CFU/g).

### Statistical Data Analysis

Statistical data analysis was performed using JMP Pro version 14.2.0 (SAS Institute Inc., Cary, NC, United States). *P*-value < 0.05 were considered statistically significant.

Linear mixed effects models were used to examine the influences of genus target (La-14 or Bi-07), assay target (GroEL or strain specific), and assay type (GroEL, strain specific, or plate count) on the cell enumeration results. In the models, cell enumeration (copies/g or CFU/g) was the dependent variable. Various fixed effect factors (assay target, genus target, dye concentration, depending on the purpose of the tests) and a random effect factor (lot), as well as their two-way interactions, were included as independent variables. Random effect factor takes inter-lot differences into consideration, while the fixed effect factors are the focus of this study. For example, to compare the efficiencies by t-ddPCR assay target, the following linear mixed effects model was constructed:

C⁢e⁢l⁢l⁢E⁢n⁢u⁢m⁢e⁢r⁢a⁢t⁢i⁢o⁢n∼I⁢n⁢t⁢e⁢r⁢c⁢e⁢p⁢t+A⁢s⁢s⁢a⁢y+L⁢o⁢t+A⁢s⁢s⁢a⁢y*L⁢o⁢t+ε

where “Assay” is a fixed effect indicator variable to specify assay target (1 if GroEL and 0 if strain specific), and “Lot” is a random effect factor. To test the significance of fixed effect factors (“Assay” in this case), likelihood ratio test was performed. If the factor is significant in the test and there are more than two levels, a *post hoc* Tukey HSD all pairwise comparison is provided.

Detailed schematic depiction of each model was listed in the subscript of Tables 2, 3. Model diagnostic plots including Q-Q plot and residual plots were used for visual inspection of model assumptions (normality, homoscedasticity, and independence).

**TABLE 2:** Results of linear mixed effects model examining the influences of assay target and genus target on the cell enumeration with Lot as a random effect factor.

**Comparison**	**Strain**	**Fixed effect**	**Regression coefficient**	**Standard error**	**95% C.I.**	***p*-value**
Pretreatment Efficiency by Assay Target^a^	La-14	Assay^a^	−4.78e10	1.73e10	(−1.22e11, 2.678e10)	0.1102
	Bi-07	Assay^a^	−5.56e10	5.5e10	(−2.92e11, 1.81e11)	0.4186
PE51 Efficiency by Genus Target^b^	La-14 vs. Bi-07	Strain^b^	−1.21e11	1.41e10	(−1.6e11, −8.15e10)	0.0010**
		Dye	−4.970e7	1.079e6	(−5.269e7, −4.671e7)	< 0.0001**
		Strain^b^ × Dye	8.879e6	1.079e6	(5.883e6, 1.187e7)	0.0012**
PE51 Efficiency by Assay Target^c^	La-14	Assay^c^	−1.36e11	1.2e10	(−1.88e11, −8.45e10)	0.0077**
		Dye	−6.785e7	1.432e6	(−7.401e7, −6e169e7)	0.0004**
		Assay^c^ × Dye	9.266e6	9.259e6	(−9.027e6, 2.756e7)	0.3185
	Bi-07	Assay^c^	−5.59e10	1.26e10	(−1.1e11, −1.863e9)	0.0469^∗^
		Dye	−4.441e7	1.989e6	(−5.294e7, −3.585e7)	0.0020**
		Assay^c^ × Dye	3.585e6	8.127e6	(−1.247e7, 1.964e7)	0.6598

^*a*^: A schematic depiction of the linear mixed effects model is Cell copies ∼ Intercept + Assay + Lot + Lot × Assay + error, where Assay is a fixed effect indicator variable (1 if GroEL and 0 if strain specific assay), and Lot is a random effect factor. ^*b*^: A schematic depiction of the linear mixed effects model is Cell copies ∼ Intercept + Strain + Dye Concentration + Lot + Lot × Strain + Lot × Dye Concentration + Strain × Dye Concentration + error, where Strain is a fixed effect indicator variable (1 if Bi-07 and 0 if La-11), Dye Concentration is a fixed effect factor, and Lot is a random effect factor. The assay used in this experiment was GroEL for both strains. ^*c*^: A schematic depiction of the linear mixed effects model is Cell copies ∼ Intercept + Assay + Dye Concentration + Lot + Lot × Assay + Lot × Dye Concentration + Assay × Dye Concentration + error, where Assay is a fixed effect indicator variable (1 if GroEL and 0 if strain specific assay), Dye Concentration is a fixed effect factor, and Lot is a random effect factor. *p < 0.05; **p < 0.01.

**TABLE 3:** Linear mixed effects model comparing v-ddPCR versus plate count, with Lot as a random effect factor.

**Strain**	***p*-value of likelihood ratio test on Assay^a^**	***Post hoc* Pairwise Comparison (Tukey HSD)**				
		**Assay**	**Difference**	**Standard Error**	**95% C.I.**	***p*-value**
La-14	0.0045**	GroEL vs. SS^b^	−2.17e11	1.454e10	(−2.86e11, −1.48e11)	0.0030**
		GroEL vs. Plate	−1.96e11	1.454e10	(−2.65e11, −1.28e11)	0.0039**
		SS^b^ vs. Plate	2.089e10	1.454e10	(−4.78e10, 8.953e10)	0.4451
Bi-07	0.0725**	GroEL vs. SS^b^	–^c^	–^c^	–^c^	–^c^
		GroEL vs. Plate	–^c^	–^c^	–^c^	–^c^
		SS^b^ vs. Plate	–^c^	–^c^	–^c^	–^c^

^*a*^: A schematic depiction of the linear mixed effects model is Cell copies ∼ Intercept + Assay + Lot + Lot × Assay + error. There are three levels in Assay: GroEL, strain specific, and plate count. Therefore, the “Assay” in the model schematic depiction is composed of two fixed effect indicator variables, GroEL (1 if GroEL, 0 if otherwise) and strain specific assay (1 if strain specific assay, 0 if otherwise). Lot is a random effect factor. Likelihood ratio test was conducted to test the fixed effect “Assay.” If it is significant, then a *post hoc* Tukey HSD all pairwise comparison is provided. The concentration used in GroEL and strain specific assays were 170 and 1400 nM for Bi-07 and La-14, respectively. ^*b*^: SS refers to strain specific assay. ^*c*^: The Likelihood ratio test on Assay was not significant, so there is no need to conduct *post hoc* pairwise comparison analysis. **p < 0.01.

## Results

### Comparison of Pretreatment Efficiency by Assay Target

**FIGURE 3:**
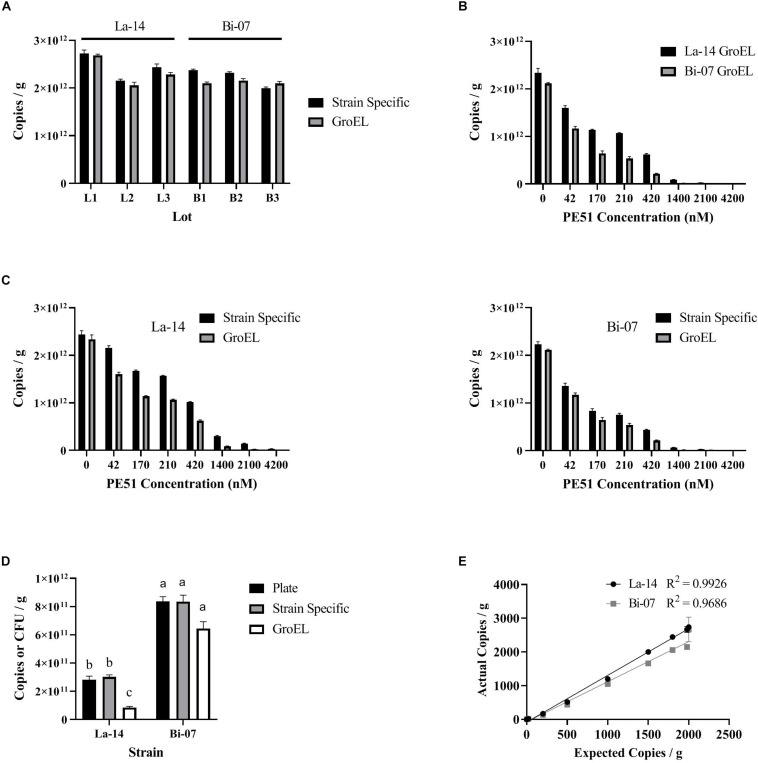
Enumeration results based on ddPCR (using GroEL or strain specific assays) and plate count. **(A)** Comparison of pretreatment efficiency by assay target; **(B)** Comparison of PE51 efficiency by genus target; **(C)** Comparison of PE51 efficiency by assay target; **(D)** Comparison of v-ddPCR and plate count results; **(E)** Comparison of expected and actual v-ddPCR results. **(A–D)** correspond to the linear mixed effects models listed in Tables 1, 2, respectively.

Before comparing v-ddPCR results based on genus targets (La-14 or Bi-07) and assay targets (GroEL or strain specific), we first enumerated La-14 and Bi-07 using t-ddPCR (no PE51 treatment) (Figure 3A), aiming to rule out the impact from the design of assays. GroEL and strain specific assays resulted in similar copies/g in La-14 and Bi-07 across six random lots, with no apparent patterns (Figure 3A). Further analysis using a linear mixed effects model taking inter-lot differences into consideration showed no statistically significant association between assay targets and enumeration results in both strains (*p*-values of fixed effect “Assay” were 0.1102 and 0.4186 for La-14 and Bi-07, respectively) (Table 2), which indicates no significant difference in t-ddPCR results using GroEL and strain specific assays.

### Comparison of PE51 Efficiency by Genus Target

To compare the PE51 efficiency by genus target, v-ddPCR was performed utilizing GroEL assays on La-14 and Bi-07 lots with PE51 treatment ranging from 0 to 4200 nM (Figure 3B). With increasing dye concentration, both assays resulted in decreased copies/g. However, Bi-07 always exhibited less copies/g than La-14 at the same dye concentration. This result can be further supported by the linear mixed effects model, which concluded a significant association between cell enumeration and genus targets (*p*-value of fixed effect “Strain” was 0.001) (Table 2). The “Strain x Dye” interaction term was also significant (*p* = 0.0012, regression coefficient = 8.879E + 6), indicating a diminished discrepancy in copies/g between the two strains as dye concentration increased.

### Comparison of PE51 Efficiency by Assay Target

To compare the PE51 efficiency by assay target, v-ddPCR was performed utilizing GroEL and strain specific assays on La-14 and Bi-07 lots with PE51 treatment ranging from 0 to 4200 nM (Figure 3C). Copies/g results using strain specific assays tended to be greater than that using GroEL assays at the same dye concentration. The linear mixed effect model built to test the influence of assay target showed a significant association between cell enumeration and assay target in both La-14 and Bi-07 (*p*-values of fixed effect “Assay” were 0.0077 and 0.0469) (Table 2), revealing the discrepancy in v-ddPCR results due to the gene targeted.

### Comparison of v-ddPCR and Plate Count Results

To compare v-ddPCR results to plate count, v-ddPCR was performed utilizing GroEL and strain specific assays with 1400 and 170 nM PE51 for La-14 and Bi-07 lots, respectively (Figure 3D and Table 3). There was no significant difference in the enumeration results based on plate count and v-ddPCR using strain specific assays in both La-14 (*p* = 0.4451 in *post hoc* Tukey HSD test) and Bi-07 (*p* = 0.0725 in likelihood ratio test). GroEL assays, however, were significantly different from plate count in La-14 (*p* = 0.0039 in *post hoc* Tukey HSD test), but not in Bi-07 (*p* = 0.0725 in likelihood ratio test).

### Comparison of Expected and Actual v-ddPCR Results

To assess the linearity of the v-ddPCR assays, actual cell enumeration results (copies/g) were compared to expected cell counts using La-14 and Bi-07 strain specific primers, over a 2-log dilution series. There was excellent linear relationship between expected and actual results (*R*^2^ = 0.9926 and 0.9686, respectively) (Figure 3E).

## Discussion

The use of a single concentration of PMA or EMA has been shown to accurately measure loss in viability when comparing different treatment groups such as untreated and heat/isopropanol killed cells (Nkuipou-Kenfack et al., 2013; Nogva et al., 2003). In contrast, several studies report the need to adjust concentration based on several factors including sample turbidity, pH, salt concentration, excess dead cells, and in accordance with the genus, species and even strain of interest (Fittipaldi et al., 2012; Gobert et al., 2018). A mixture of PMA and EMA has been purposed as an improvement to the use of either dye alone due to its ability to distinguish between live and dead cells based on both membrane integrity (PMA) and metabolic ability (EMA) (Codony et al., 2015).

A mixture of PMA and EMA (PE51) was investigated, at specified concentrations, to exclude DNA from freeze dried probiotic products. PE51 efficiency was evaluated across the two most common probiotic genera, *Bifidobacterium* and *Lactobacillus* when a common DNA target was utilized, and when utilizing two different assay targets within the same strain. Lastly, we investigated the potential of v-ddPCR assays, when paired with PE51, to accurately enumerate freeze dried probiotic products.

The ability of PE51 to exclude DNA from freeze dried probiotics, PE51 efficiency, was analyzed over a 2-log concentration gradient. Significant exclusion capacity was observed on all assays, even at the lowest concentrations tested (42 nM), much lower than the concentrations suggested by the manufacturer or as reported in previous literature (50 μM/100 μM) (Biotium, 2019; Nocker and Camper, 2006). Significant differences continue to be observed at higher concentrations with results dropping well below viable counts obtained through plate count enumeration (Figure 3C). This concentration dependent exclusion agrees with previous literature on PMA and EMA (Kobayashi et al., 2009; Kramer et al., 2009; Nogva et al., 2003; Oketiè et al., 2015; Yáñez et al., 2011) and calls into question protocols, viability kits and other products which recommend the use of a single concentration of dye for distinguishing viable cells and instead supports the concept of dye optimization.

To investigate the relationship between PE51 efficiency and the genus of an organism, ddPCR assays targeted a highly conserved gene, GroEL (Zeilstra-Ryalls et al., 1991), for two probiotic strains, La-14 a *Lactobacillus* and Bi-07 a *Bifidobacterium*. Despite similar pre-viability treatment efficiency as measured by t-ddPCR and averaging approximately 200% higher viability when measured by plate count enumeration (La-14 = 2.81E + 11, Bi-07 = 8.37E + 11), Bi-07 exhibited significantly lower v-ddPCR results when compared to La-14 across the range of PE51 concentrations analyzed (Figure 3B). These data suggest the need to optimize PE51 concentration based on the genera of the bacteria being analyzed, even when a common assay target is utilized. In agreement with these findings, different viability outcomes have been reported at the species level when utilizing EMA with identical primer sets, which has been suggested to be associated with physiological differences such as membrane permeability and efflux efficiency (Fittipaldi et al., 2012; Kobayashi et al., 2009; Nocker et al., 2006).

The relationship between PE51 efficiency and assay choice was also investigated. To do so, a number of potential variables were controlled, first physiological differences were controlled by utilizing two assays to analyze the same strain of bacteria, La-14 or Bi-07. Next, pretreatment efficiency was controlled through design considerations; GroEL assays were designed to have similar characteristics to strain specific counterparts, while targeting a different gene. Similar pretreatment efficiency was observed as measured by t-ddPCR (Figure 3A). Finally, the treatment processes were controlled by performing the enumeration with both assays on the same treated samples.

Surprisingly, v-ddPCR results indicated a significant difference for both La-14 and Bi-07 when PE51 treated samples were enumerated utilizing GroEL or strain specific targets (Table 2). This difference suggests the need for optimization of PE51 concentration at the assay level. While the cause for these observations is not well understood, previous research has suggested sequence specificity and secondary nucleic acid structures may affect the binding efficiency of DNA intercalating dyes (Hardwick et al., 1984; Parshionikar et al., 2010). One proposed solution to this problem is the use of longer target sequences (Soejima et al., 2011), however, the use of long sequence ddPCR assays has not been widely studied for probiotics and may reduce specificity and limit other assay design considerations.

Viability of probiotics is a topic of great debate, but plate count enumeration has a long history of use in formulation of products, quality measures, clinical dosages, and research and development efforts (Fenster et al., 2019). Rapid methods utilizing viability dyes used in too high or too low a concentration have been shown to under or overestimate viable counts (Løvdal et al., 2011; Nkuipou-Kenfack et al., 2013). For these reasons, we find that the use of alternative methods that reproduce plate count results are of greater value to the probiotic industry. Based on this assessment v-ddPCR with optimized PE51 concentrations and plate count enumeration results were evaluated for agreement. Here, the optimized strain specific v-ddPCR assays for both La-14 and Bi-07 were demonstrated to agree with plate count enumeration results (Table 3). Additionally, these assays were assessed for linearity using three commercial lots diluted over a 2-log concentration gradient and were found to have excellent linear relationship. In agreement with previous literature, we have demonstrated the ability of viability dyes (PMA, EMA, PEMAX, and PE51), paired with PCR based methods (qPCR, cdPCR, and ddPCR) to meet this criterion (Hansen et al., 2018; Hansen et al., 2020; Meng et al., 2010; Morovic et al., 2018).

In conclusion, the use of PE51 for the detection of viable probiotic cells confirms differences in dye efficiency between genera and builds upon previous research findings to add assay target to the level of specificity required when optimizing viability treatment. Here we discussed the caution that must be taken when utilizing a single concentration of viability dye, the need to optimize viability assays, and the ability of v-ddPCR to agree with traditional plate count enumeration results. Investigation into the use v-ddPCR methods on blended probiotic products will lead to strain specific enumeration capabilities currently lacking in traditional plate count methods. Further research to elucidate the need for dye concentration optimization will aid in the creation of more robust and universal methods, however, large scale data collection is needed to establish a history of testing, facilitate new assay development and generate methods better suited for the future of the probiotic industry. Based on improvements offered by v-ddPCR, which are well documented to include increased precision, specificity and decreased time to results, we suggest the implementation of v-ddPCR into current probiotic product testing.

## Data Availability Statement

All datasets presented in this study are included in the article/Supplementary Material.

## Author Contributions

AK and SA managed the ideation and study design. PT performed statistical analysis. AK, SA, PT, and VF were responsible for interpretation of the results. CW was the project manager and contributed to the ideation and experimental design. All authors contributed to writing and editing the manuscript.

## Conflict of Interest

The authors declare that this study received funding from DuPont Nutrition & Biosciences. The funder had the following involvement in the study: The microbial strains used are commercially sold by DuPont Nutrition & Biosciences, and DuPont Nutrition & Biosciences reviewed the manuscript prior to submission. All authors are employees of DuPont Nutrition & Biosciences.
